# Plant-Derived Products with Therapeutic Potential against Gastrointestinal Bacteria

**DOI:** 10.3390/pathogens12020333

**Published:** 2023-02-15

**Authors:** Fatimah I. Qassadi, Zheying Zhu, Tanya M. Monaghan

**Affiliations:** 1 School of Pharmacy, University of Nottingham, Nottingham NG7 2RD, UK; 2 Department of Pharmacognosy, College of Pharmacy, Prince Sattam bin Abdulaziz University, Al-Kharj 11942, Saudi Arabia; 3 NIHR Nottingham Biomedical Research Centre, University of Nottingham, Nottingham NG7 2RD, UK; 4 Nottingham Digestive Diseases Centre, School of Medicine, University of Nottingham, Nottingham NG7 2RD, UK

**Keywords:** plant-derived products, antibiotic resistance, new drug targets, gastrointestinal infections, clinical trials

## Abstract

The rising burden of antimicrobial resistance and increasing infectious disease outbreaks, including the recent COVID-19 pandemic, has led to a growing demand for the development of natural products as a valuable source of leading medicinal compounds. There is a wide variety of active constituents found in plants, making them an excellent source of antimicrobial agents with therapeutic potential as alternatives or potentiators of antibiotics. The structural diversity of phytochemicals enables them to act through a variety of mechanisms, targeting multiple biochemical pathways, in contrast to traditional antimicrobials. Moreover, the bioactivity of the herbal extracts can be explained by various metabolites working in synergism, where hundreds to thousands of metabolites make up the extract. Although a vast amount of literature is available regarding the use of these herbal extracts against bacterial and viral infections, critical assessments of their quality are lacking. This review aims to explore the efficacy and antimicrobial effects of herbal extracts against clinically relevant gastrointestinal infections including pathogenic *Escherichia coli*, toxigenic *Clostridioides difficile*, *Campylobacter* and *Salmonella species*. The review will discuss research gaps and propose future approaches to the translational development of plant-derived products for drug discovery purposes for the treatment and prevention of gastrointestinal infectious diseases.

## 1. Introduction

Globally, gastrointestinal (GI) infections are the second most prevalent infectious diseases after respiratory infections +. Bacterial gastroenteritis and diarrhoea are commonly caused by specific pathogenic strains of *Escherichia coli*, *Campylobacter, Enterococcus faecalis*, *Salmonella* spp., *Shigella*, and *Clostridioides difficile* [1,2,3]. Contaminated food and water and inadequate hygiene are key factors in the transmission of GI infections [4]. The most common GI symptom is diarrhoea, which is usually self-limiting and typically resolves within a few days for most healthy individuals. If diarrhoeal disease progresses, young children and older adults with co-morbidities may succumb to excessive dehydration, malnutrition, bacteremia, and other complications, which may result in death [5]. The World Health Organization (WHO) has reported more than 1.7 billion diarrhoeal disease cases each year. In addition, diarrhoeal diseases were responsible for the deaths of 370,000 children under five years of age in 2019 [4]. Identifying the aetiology of infectious gastroenteritis and making appropriate treatment decisions are crucial for patient management.

The search for new antimicrobial agents has intensified in recent years due to bacterial resistance to current antibiotics, with natural products playing an integral role [6]. Additionally, the lengthy testing process for new drugs before they are approved for commercialization adds to the call for alternative therapeutic approaches. [7]. Several methods have been proposed to investigate the antibacterial properties of natural products. The ancient world has used plants as medicaments for thousands of years, especially in Asian regions like India, China, and Japan, and in some African nations [8]. Among tribal people, these plants are mostly used as folkloric remedies mainly due to their ease of availability and relatively low prices [9,10]. According to a study examining the use of complementary and alternative medicine (CAM) in patients with GI disorders, nearly half were using herbal treatment, acupuncture, or mind/body treatments [11]. Several plant-derived products with various chemical structures have been discovered based on traditional knowledge of their medicinal uses [12]. The structural diversity of phytochemicals enables them to act through a variety of mechanisms (targeting multiple biochemical pathways), in contrast to traditional antibiotics [13,14]. Despite a significant increase in publications on this topic, the mechanisms of action of herbal extracts have remained elusive. In this review, we will discuss the preclinical and clinical evidence for the use of herbal-derived products as antibacterial agents for the treatment of clinically relevant infectious diseases associated with the GI tract (GIT).

## 2. Proposed Mechanisms of Action of Plant-Derived Products

Phytochemicals show promise in supporting and maintaining GI health (including the management of diarrhoea, gastritis, and gastric ulcers), and combating bacterial infections. There are thousands of structurally different compounds produced by plants (polyphenols, terpenoids, phenolic acids, essential oils, lectins, polypeptides, and alkaloids), each possessing different biological properties, which provide the scientific basis for the use of herbs in traditional medicine in many ancient communities [15]. Most of these compounds are secondary metabolites, which are heterogeneous from both a biosynthetic and structural perspective. The presence/absence of some specific functional groups increases the diversity of compounds belonging to the same biosynthetic class [16].

**Figure 1:**
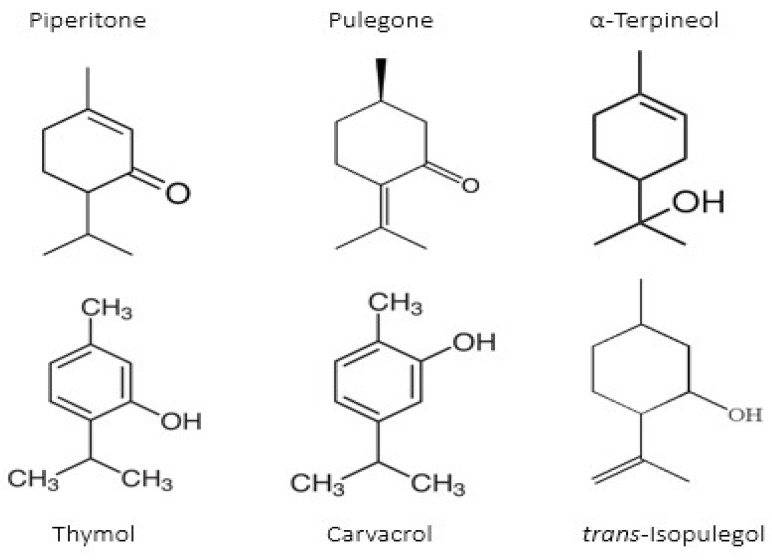
Chemical structures of *P*-Menthane type monoterpenoids.

A great example of the strong relationship between the molecular structure of a compound and its possible biological properties can be found in the *p*-menthane type monoterpenoids Figure 1. The differences between these molecules, even if they appear insignificant at first glance, can be very important when considering the biological properties of these compounds, even if some are only mutually isomeric, differ in opposition to double bonds, and others have different functionalities/hetero atoms [12]. As an example, carvacrol, a compound isomer to thymol, was found to have antimicrobial effects primarily via cytoplasmic membranes, where the effect of carvacrol is enhanced by its hydroxyl group, which acts as a transmembrane carrier of monovalent cations, disrupting the membrane potential [17,18], while thymol, another aromatic p-menthane type monoterpene phenol, is thought to interact with both inner and outer membranes of the cell by integrating in the polar headgroup region of the lipid bilayer, increasing its permeability [19].

**Figure 2:**
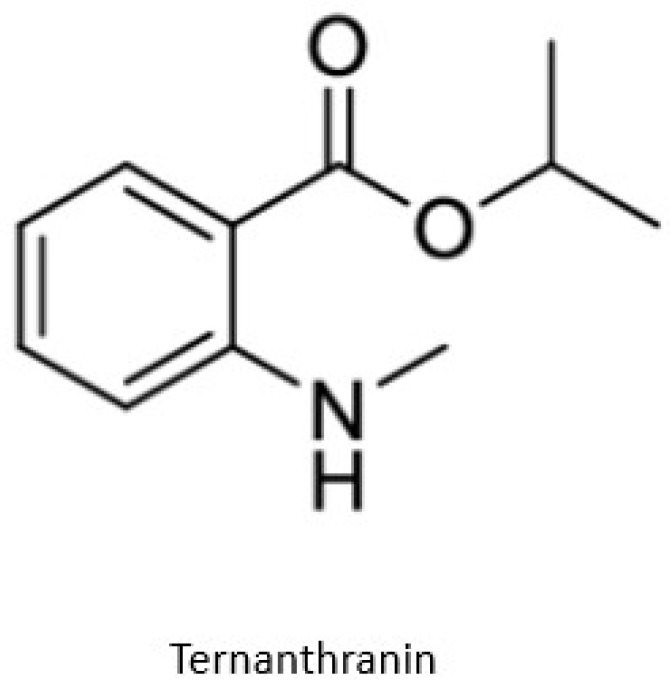
Chemical structure of Ternanthranin (*N*-methyl derivative of anthranilic acid), an alkaloid constituent of Mexican orange blossom, has anti-inflammatory and antibacterial activities [20].

Ternanthranin, an alkaloid found in *Choisya ternata* Kunth. essential oil (Mexican orange blossom), is not only structurally similar to aspirin (acetylsalicylic acid), but also pharmacologically similar (*N*-methyl derivative of anthranilic acid) (Figure 2) [20]. Interestingly, anthranilic acid derivatives are considered to be important drug discovery pharmacophores [21] Many of these compounds are currently in use, including mefenamic acid and meclofenamates, which have analgesic, anti-inflammatory, and antipyretic properties [21,22].

In general, antibacterial actions of action of plant-derived products include inhibition of bacterial cell wall synthesis, cell membrane destruction, inhibition of bacterial protein synthesis, DNA replication, and metabolic pathways, making them excellent candidates for future drug development [6,15,23,24]. Additionally, they can inhibit mechanisms involved in bacterial resistance to antibiotics, including overexpression of efflux pumps, structural modification of porins, and antibiotic-target modification, which may lead to new approaches to overcoming antibiotic resistance [6,15]. Some plant-derived compounds with antibacterial properties against clinically relevant GI bacteria and their mechanism of actions are illustrated in Table 1.

Enterohemorrhagic *Escherichia coli* (EHEC), specifically, *E. coli* O157:H7, is one of the most important serotypes among the different *E. coli* pathogenic groups, which include enterotoxigenic (ETEC), enteropathogenic (EPEC), enterohaemorrhagic (EHEC), enteroinvasive (EIEC), diffusely adherent (DAEC), and enteroaggregative *E. coli* (EAEC) [45]. It is estimated that these serotypes cause 73,000 illnesses annually and 60 deaths in the United States alone [46]. Plant-derived products used for treating these pathogens possess antibacterial activities through inhibition of toxic production and toxin-mediated cellular damage on host cells [33].

Ginger (*Zingiber officinale* Roscoe) is one of the most used spices. Zingerone (vanillylacetone) is reported to be the active constituent responsible for the antidiarrhoeal efficacy of ginger [27]. Zingerone reduced heat-labile enterotoxin (LT)-induced diarrhoea in ETEC through blocking the binding to GM1 ganglioside receptors, thus preventing accumulation of diarrhoeal fluids in vivo [27]. The FDA recognizes ginger as a safe herb, and German monographs report no known side effects or drug interactions with ginger [27]. Similarly, extracts of Chinese medicinal herbs, *Rhus chinensis* Mill (Chinese sumac or nutgall tree), and Indian medicinal plant, *Berberis aristata* DC (Indian barberry or tree turmeric), were effective in preventing ETEC heat-labile enterotoxins from binding to their surfaces [47]. Gallic acid, the major component of *Galla chinensis*, was effective for treating LT-induced diarrhoea by causing inactivation of ETEC enterotoxins [47]. *Galla chinensis* is gall caused by the Chinese aphid (family Pemphigidae) on the Rhus leaves of the family Anacardiaceous (mainly, *Rhus chinensis* Mill) [48]. Furthermore, large amounts of tannins present in *Galla* chinensis inhibit intestinal bacterial growth in vitro including *Clostridium perfringens*, *Clostridium paraputrificum*, and *E. coli* without affecting the growth of the intestinal probiotics (*bifidobacterial* and *lactobacilli*) [49]. It was reported that tannins extract from *Galla chinensis* was one of the active compounds to resist *E. coli* and protect mice from infection by *Enterotoxigenic E. coli* [50].

*Camellia sinensis* tea leaves contain galloylated tea catechins, which have been observed to act at the level of the bacterial cell membrane level [51,52] inducing perturbations of the ordered structure of bilayers of phosphatidylcholine and phosphatidylethanolamine found in bacterial cell walls [51]. Epigallocatechin-3-gallate (EGCG), a major polyphenolic compound extracted from green tea leaves, was the most effective catechin causing inhibition of the growth of *E. coli* cells by damaging the lipid bilayer membrane. These results suggest a strong effect of catechins from green tea on bacterial phospholipid synthesis [53]. Furthermore, a decrease in Vero toxins (VTs) production in pathogenic EHEC has been observed following treatment with catechin and epigallocatechin in green tea. At concentrations of 0.05 mg/mL, these compounds markedly inhibit the release of extracellular VT into culture supernatant fluid [28].

Eugenol, the major component of clove (*Syzygium aromaticum* (L.) Merr. & L.M. Perry.), inhibits the production of two verotoxins (VT1 and VT2) in EHEC by modulating the transcription of *stx 1* and *stx 2*, respectively [33]. *Stx1* and *Stx2* gene expression levels were similarly reduced with sub-inhibitory concentrations of *Curtisia dentata* extract, trans-cinnamaldehyde (major component of cinnamon) and carvacrol (obtained from oregano oil) [33,54]. These studies suggest that plant-derived compounds affect not only toxin-receptor binding, but also toxin genes and host receptor expression, reducing toxin-mediated pathology.

Proanthocyanidins show antibacterial activity against *E. coli*, the leading cause of bacteria-mediated gastrointestinal infections, by reducing bacterial adhesion to the gastrointestinal mucosa [55]. These phenolic compounds are produced in relatively high concentrations in *Vaccinium macrocarpon* (cranberry) and show promising activity against bacterial virulence toxins and biofilm formation [25,56]. Although the activity of phenolics is generally weak and nonspecific, they target Gram-positive, rather than Gram-negative bacteria [29,57].

An alkaloid, berberine, derived from the rhizomes of *Berberis vulgaris*, exhibits antibacterial properties against a wide range of pathogenic bacteria either alone or in combination with antibiotics [58,59]. Berberine was effective in targeting proteins responsible for upholding the structure of cells and for cell division. This led Domadia, et al. [60] to examine the effect of berberine on filamenting temperature sensitive mutant Z (FtsZ) activity, a bacterial cytokinesis protein essential for bacterial cell division. In silico modelling suggested that the hydrophobic residues in the GTP binding region may facilitate berberine binding to FtsZ, which makes it effective towards Gram-negative bacteria [61]. Scanning electron microscopy revealed that berberine altered bacterial morphology and ruptured cells, causing leakage of intracellular substances and the release of more K+ and Ca+, which inhibited protein synthesis [62]. Furthermore, this compound could decrease the activity of the Na+/K+-ATPase in cell membranes. Due to these effects, berberine prevents bacterial proteins from being expressed by destroying the cell membrane structures, ultimately resulting in the death of bacteria [60,62]. Interestingly, berberine, an efflux pump substrate, was shown to reduce the resistance of pathogenic strains to conventional antibiotics when it is co-administered with different efflux pump inhibitors [63]. Through inhibition of the MexXY-OprM efflux pump system, berberine exhibits synergistic effects with carbapenem in re-sensitizing imipenem-resistant *Pseudomonas aeruginosa* [64].

The mechanism by which berberine treats diarrhoea caused by *V. cholerae* and *E. coli* has been extensively investigated. Berberine directly inhibited *V. cholerae* and *E. coli* enterotoxins in vitro [32]. The inhibition of *E.coli* biofilm by berberine was associated with the suppression of quorum-sensing genes such as *pfS*, *hflX*, and *fliA* [65]. In addition, berberine significantly inhibited the secretion of *E. coli* heat-stable enterotoxins in a mouse model [32]. Even though the mechanism of action of the drug is not yet fully known, these data support its clinical value in treating acute diarrhoea [32]. Berberine significantly decreased the expression levels of the ATP-binding protein of the lipoprotein release system (LolD) and the binding protein of the phospholipid ABC transporter (MlaB) [66]. The absence of LolD decreases lipoprotein transport to the outer membrane of bacteria [67]. Additionally, MlaB contributes to maintaining outer membrane lipid asymmetry through the transport of aberrantly localized phospholipids between the outer and inner membranes, while Mla plays an important role in transporter assembly and function [68]. In Gram-negative bacteria, lipid asymmetry is crucial to the outer membrane’s function as a selectively permeable barrier. In the absence of LloD and MlaB expression, cell membrane stability is weakened [66] Berberine can significantly inhibit bacterial cell wall synthesis in *E.coli* by downregulating the capsule synthesis promoter (RcsF) and Undecaprenyl-diphosphatase (YbjG) [66]. Both are involved in bacterial cell wall synthesis, and the latter is necessary to synthesize undecaprenyl phosphate, a C55 lipid carrier for cell wall synthesis [69,70]. Consequently, incomplete cell wall synthesis reduced the antibiotic resistance of *E. coli* [32]. Even though the mechanism of action of the drug is not yet fully known, these data support its clinical value in treating acute diarrhoea [32]. Berberine significantly decreased the expression levels of the ATP-binding protein of the lipoprotein release system (LolD) and the binding protein of the phospholipid ABC transporter (MlaB) [66]. The absence of LolD decreases lipoprotein transport to the outer membrane of bacteria [67]. Additionally, MlaB contributes to maintain outer membrane lipid asymmetry through the transport of aberrantly localized phospholipids between the outer and inner membranes, while Mla plays an important role in transporter assembly and function [68]. In Gram-negative bacteria, lipid asymmetry is crucial to the function of the outer membrane as a selectively permeable barrier. In the absence of LloD and MlaB expression, cell membrane stability is weakened. [66] Berberine can significantly inhibit bacterial cell wall synthesis in *E.coli* by downregulating the capsule synthesis promoter (RcsF) and Undecaprenyl-diphosphatase (YbjG) [66]. Both are involved in bacterial cell wall synthesis, and the latter is necessary to synthesize undecaprenyl phosphate, a C55 lipid carrier for cell wall synthesis [69,70]. Consequently, incomplete cell wall synthesis reduced the antibiotic resistance of *E. coli* [66].

Aromatic essential oil constituents such as carvacrol (2-methyl-5-(1-methylethyl)-phenol) and cinnamaldehyde (3-phenyl-2-propenal) possess broad antimicrobial activity that extends to food spoilage and pathogenic microorganisms, including drug-resistant and biofilm-forming microorganisms [33,34]. The antibacterial activity of these compounds is due to the considerable changes in the structural and functional properties of cytoplasmic membranes [19,71]. An in vitro study found that carvacrol and cinnamaldehyde reduce production of *C. difficile* toxins A (TcdA) and toxin B (TcdB) and toxicity while maintaining normal gut flora growth [34]. These compounds were found to down-regulate toxin production genes. Several toxin repressor genes were found to be positively modulated by the compounds as well, potentially through global pleiotropic repressor CodY [34,72]. As a result of carvacrol supplementation, diarrhoea incidence in mice was significantly reduced and *C. difficile*-induced clinical symptoms were mitigated, causing a favourable shift in gut microbiota composition without negatively affecting gut microbiome diversity [73]. Based on these findings, carvacrol may be a potential anti-*C. difficile* agent, but further clinical studies are needed.

Several studies have shown that allicin in garlic juice (*Allium sativum* L.) inhibits *C. difficile* toxin production (TcdA) along with the genes encoding TcdR, an RNA polymerase sigma factor needed for maximal expression of TcdA (4.7 mg/mL) [74,75]. The antibacterial activity of garlic may also be attributed to other compounds such as saponins and flavonoids. A study found that the ginger component zingerone protected cells against *C. difficile* toxins TcdA and TcdB by blocking either the toxin-binding site on toxin molecules or the host cell receptors (0.3 mg/mL) [75]. Further investigation of biologically active components showed that zingerone was the active constituent responsible for the anti-diarrhoeal effect of ginger [27]. In an earlier study, Doughari et al. investigated the effect of onion bulb extract and its bioactive components on inhibition of toxigenic *C. difficile* toxins TcdA and TcdB in vitro. Fresh onion bulb extract (12.5% *v*/*v*) significantly decreased toxin production and activity. The primary mechanism of action of this extract was the inhibition of *C. difficile* sporulation at different stages [57]. In a mouse model of *C. difficile* infection (CDI), berberine (100 mg/kg/day) combined with vancomycin was more effective than vancomycin alone. Treatment with berberine prevents CDI relapses and reduces mortality significantly [76].

A novel lead compound, chromomycin A2/NP-00387, with potent anticlostridial activity in vitro and in vivo, conferred 100% protection to infected mice against clinical manifestations of CDI at a concentration of 0.03 μg/mL [77]. The compound belongs to the aureolic acid family of bacterial natural products, and exhibits time-kill kinetics similar to fidaxomicin [77]. Furthermore, chromomycin A2 has been shown to be non-toxic to Caco-2 cells and to be as effective as vancomycin in protecting mice against *C. difficile* [78]. It will be necessary to conduct further trials to evaluate its efficacy in ameliorating CDI symptoms, including validating its mechanism of action and determining its efficacy in a recurrent infection model.

*Scutellaria baicalensis* Georgi. (Chinese skullcap) roots contain baicalin (5,6,7-trihydroxyflavone), a major flavone glycoside identified in the Chinese Pharmacopoeia as a medicinal herb [37]. Researchers have observed that baicalin was capable of significantly inhibiting *H. pylori* in the murine stomach [35]. Previously, researchers have identified that baicalin improved renal function and significantly reduced Shiga-Like Toxin 2 lethality in mice. Additionally, structural and biophysical studies have shown that baicalin binds directly to *Stx* to inactivate the toxin and promote toxin oligomerization [36]. Moreover, the same research group challenged mice with Enterohemorrhagic *E. coli* O157:H7 and showed that baicalin reduced lethality in mice, but its exact mechanism of action remains unclear [38].

There are only a few reports available in which researchers have assessed essential oils [39,79,80] and fruits extract [81] against *C. difficile*. A recent study examined the effect of essential oil compounds on mixed human faecal microbiota. As a result of the study, thymol and geraniol at a concentration of around 100ppm may be effective in suppressing pathogens in the small intestine without damaging beneficial commensal bacteria of the colon [39]. Several of the fatty acids found in virgin coconut oil have been tested for their antimicrobial properties against *C. difficile* in vitro [79]. Lauric acid (C12) significantly inhibited the growth of the bacteria. At 2 mg/mL of lauric acid exposure, transmission electron microscopy clearly demonstrated that both the cell membrane and cytoplasm were disrupted. Furthermore, live/dead staining of bacterial cells following treatment with selected fatty acids confirmed the physiological changes in bacterial cell membrane integrity [79].

It has become increasingly difficult to manage campylobacteriosis because of antibiotic-resistant strains of Campylobacter. Campylobacter has been reported to possess a wide variety of cytotoxic activities [82], but only the cytolethal distending toxin (CDT) has been clearly defined. There is evidence that certain strains of *Campylobacter jejuni* that cause dysentery-like illness are more cytotoxic than other Campylobacter species [83]. Although some plants have been evaluated for their ability to inhibit *Campylobacter jejuni* and *Campylobacter coli*, little is known about their effect on virulence factors of these bacteria [84]. A preliminary analysis showed 21 of the 28 plant extracts inhibited Campylobacter growth, 11 of which showed a high level of inhibition (inhibition zone of 2 to 4 cm) [85]. The four plants with the best anti-Campylobacter activity were Acacia *farnesiana*, *Artemisia ludoviciana*, *Opuntia ficus-indica*, and *Cynara scolymus*. It has also been reported that Japanese green tea extracts show anti-*Campylobacter jejuni* activity [86]. A critical step in Campylobacter pathogenesis is adhesion to intestinal mucosa [82,87]). In a study by Lengsfeld, et al. [88], okra extracts inhibited the adhesion of *C. jejuni*, with a strong antiadhesive effect in vitro, but not in vivo. Similarly, extracts of *A. farnesiana*, *A. ludoviciana*, *C. scolmymus,* and *O. ficus-indica* significantly reduced Campylobacter adhesion to Vero cells [85]. This inhibitory activity was caused by flavonoids and polyphenols. It’s thought these compounds work as antimicrobials because they form a complex with extracellular and soluble proteins, which are then able to bind to bacterial cell walls [89]. Furthermore, evidence indicates that lipophilic flavonoids can disrupt microbial membranes [12,15].

## 3. Preclinical and Clinical Studies of Herbal Products in the Management of GI Infectious Diarrhoeal Disorders

The global healthcare system is heavily burdened by GIT disorders. Globally, diarrhoeal-inducing GIT disorders are one of the leading causes of death [90]. Even though pharmaceutical drugs are effective in treating most GIT ailments, a growing trend has been observed in the use of traditional treatments (e.g., through herbal remedies) [91].

Due to the absence of significant pre-clinical and clinical research on the effectiveness of herbal medicine, it is important to gather whatever scientific data to support their use in treating different pathologies. A large proportion of herbal medicines are used in developing countries [11]. There are many biologically active compounds in the natural products derived from medicinal plants. Globally, there are approximately 500,000 species of plants, but only 1% have been examined for novel bioactive compounds, and only a small fraction has been tested in randomized controlled trials as illustrated in Table 2 [92,93].

In India, raw baobab fruit (*Adansonia digitata)* is eaten to treat diarrhoea and dysentery. According to a clinical study conducted in Senegal comparing *Adansonia* fruit with oral rehydration therapy, the two treatments did not differ significantly in terms of diarrhoea duration and weight gain [94]. It is possible that the fruit’s astringent properties explain its medicinal properties, although high levels of tartaric acid can cause gastrointestinal irritation if consumed in large quantities [95].

Guava is well-known for treating diarrhoea in tropical countries through ingesting teas, decoctions, or macerations [96]. Guava leaves, *Psidium guajava* (myrtaceae), originate from Central and South America where this tree forms part of the pantropical region. Additionally, the plant can be used to treat coughs, diabetes, dysentery, fevers, leukorrhea, rheumatism, toothaches, and wounds [97]. The use of decoctions of dried leaves and aqueous extracts of leaves to treat diarrhoea has been supported by independent experiments in mice. A mixture of flavonoids including quercitrin, kaempferol, avicularin, and guaijaverin is believed to be the active components [98]. Guava has been evaluated using various in vivo models of infectious diarrhoea. An ethanol extract of *P. guajava* was shown to clear infection faster after 19 days in mice infected with *Citrobacter rodentium* [99], and mice infected with *V. cholerae* had intestinal ameliorative effects after a 4-h infection when treated with 250 mg/kg of ethanol extracts of *P. guajava* leaves [100]. A randomized double-blind clinical trial was conducted to evaluate the efficacy of the phytodrug QG5^®^, which contains *P. guajava* leaves with high flavonoid concentrations, on patients with infectious gastroenteritis. The duration of abdominal pain was significantly reduced by QG5^®^ in these patients without any serious side effects [101]. The literature lacks sufficient information on the toxicological effects of *P. guajava* extracts. According to one study, oral administration of 5000 mg/kg body weight of methanolic extract of *P. guajava* bark for 28 days did not cause toxicity in Wistar rats [102], although in histopathological analyses, repeated doses exceeding 1000 mg/kg b.w. resulted in minor liver inflammation in females treated at high doses, and a sensitivity to the extract was observed in the males [102]. Early clinical trials showed that berberine tannate combined with sulfadimidine and neomycin were effective in treating acute infective diarrhoea in children [103,104]. Among the bacteria isolated from the stools of paediatric patients, *E. coli* was most prevalent. Clinical trials conducted on 100 gastroenteritis patients showed that berberine tannate was more effective than conventional antibiotics in treating diarrhoea [105]. When berberine tannate was administered to children with acute diarrhoea, their recovery was faster than with standard antibiotic therapy [106]. Many diarrhoea-causing bacteria, such as *E. coli*, *Shigella* spp., and *Salmonella* spp. were isolated from the patients [106].

In a randomized controlled trial, berberine sulphate reduced mean stool volume in 165 adult patients with enterotoxigenic *E. coli* and *V. cholerae* diarrhoea during three consecutive 8-h periods after treatment. There have been no reported side effects associated with berberine [107]. There are probably several mechanisms underlying berberine’s anti-diarrhoeal activity, including the inhibition of intestinal secretion caused by microbial enterotoxins [32], inhibition of gastrointestinal motility [108,109], enhanced water and Na+ absorption [110], and its direct antimicrobial properties. It is still noteworthy that Minimum Inhibitory Concentrations (MICs) of berberine for bacteria associated with diarrhoea, such as *Bacillus cereus*, *E. coli*, and *V. cholerae*, present at relatively high levels (12.5–469 μg/mL) [31,111].

In a study of 50 patients with CDI and non-*C. difficile* antibiotic-associated diarrhoea, berberine cure rates were 84 and 92 percent, respectively [112]. A meta-analysis has concluded that traditional Chinese medicine such as berberine, alone or combined with Western medicine, is superior to Western medicine alone in treating antibiotic-associated diarrhoea [113]. The inhibition of *C. difficile* toxin genes was reported by Shu et al. [114] to be a potential pharmacological mechanism of traditional Chinese medicine for the treatment of CDI. Studies have shown that berberine may induce apoptosis and adversely affect liver function in vivo and in vitro [115]. Aside from gastric troubles, berberine toxicity also causes hepato- and haematotoxicity, haemorrhagic inflammatory consequences, and immune cell damage [115,116]. The literature reveals, however, that berberine’s efficacy data are far more abundant than its toxicity data [64,117,118,119]

As part of a parallel, double-blind, randomized trial of 133 European tourists in West Africa and 112 American students in Mexico, bismuth subsalicylate significantly shortened travellers’ diarrhoea duration at both study sites [120]. Bismuth subsalicylate was found to be effective and well-tolerated in an open-label, two-day study in 94 adult students with acute diarrhoea [121]. The use of bismuth subsalicylate led to the clearance of pathogenic *E. coli* in all cases and was well tolerated with no reported adverse effects. In a placebo-controlled randomized trial conducted with 252 children, 150 mg of bismuth subsalicylate per kilogram of body weight per day decreased the duration of acute diarrhoea. *C. jejuni,* enteropathogenic *E. coli*, *Salmonella* spp., *Shigella* spp., and *Vibrio* spp. were found in stool samples collected at the beginning of the study [122]. There was an in vitro inhibitory effect of bismuth subsalicylate against *C. difficile*, enterotoxigenic *E. coli, S. enterica* subsp. enterica serovar Typhimurium, and *Shigella sonnei*. Bismuth subsalicyclate was found to have MICs of 2, 8, 4–8, and 2–8 μg/mL, respectively [123]. Despite its therapeutic efficacy, Chowdhury and his team observed a significant increase in weight gain in patients treated with bismuth subsalicylate compared with placebo, without major toxicity [124] The use of over-the-counter bismuth subsalicylate for long periods of time has been reported to cause acute toxicity manifested as nephrotoxicity [125].

Several dietary supplements, herbal medicines, and over-the-counter pharmaceuticals are available on the international market that support and maintain GI health (including diarrhoea, gastritis, and gastric ulcers) [126]. Various forms are available, including capsules, liquids, tablets, and tinctures. In addition, plant extracts and derivatives containing antibacterial properties are available commercially on the international market [127]. There are various food and herbal supplements available in Europe containing extracts from roots and rhizomes of *Coptis* spp., *Berberis* spp., and *Hydrastis canadensis* as capsules, tablets, and tinctures (e.g., Huang Lian tincture (*Coptis chinensis* extract), Napiers, Edinburgh, UK; Berberine HCL 97% Capsules, British supplements, Milton Keynes, UK; and *Hydrastis Canadensis* capsules, Alfa herbal, Barcelona, Spain). In addition to supporting digestive function, these supplements are also used to treat gastrointestinal disorders, including diarrhoea [59,110]. Most clinical data on their anti-diarrhoeal effects are based on berberine, an isoquinoline alkaloid (especially in its hydrochloride, sulphate, and tannate forms) [58,104]. In Asia, berberine hydrochloride tablets are sold for the treatment of intestinal infectious diseases and diarrhoea (Northeastern Pharmaceutical Group, Shenyang, China). Furthermore, Bismuth subsalicylate (the Procter & Gamble Company, Cincinnati, OH, USA) is available in the form of liquids or chewable/swallowable tablets for the treatment of stomach and GI discomfort, including diarrhoea, indigestion, heartburn, and nausea, under the brand name Pepto-Bismol [122,123]. In Latin America, QG5 is sold to relieve inflammation, spasms, and abdominal pain, containing 166 milligrams of Psidium guajava leaves dry extract, which contains flavonoids like quercetin [101].

**Table 2:** Herbal-derived products used for treating GI infections in clinical trials.

Disease	Treatment	No. of Patients	Study Design	Dosage/Duration	Outcomes	Reference
Diarrhoea-predominant irritable bowel syndrome	(i) Ayurvedic herbal compound: *Murraya koenigii* (curry), *Punica granatum* (pomegranate), and *Curcuma longa* (turmeric) (ii) Placebo	22	Double-blind, cross-over randomized clinical trial	Twice daily/4 weeks followed by a one week wash out period	No significant improvement in the symptoms of diarrhoea-pre-dominant irritable bowel syndrome compared to placebo.	[128]
Diarrhoea-predominant irritable bowel syndrome	(i) Traditional Chinese medicine containing 11 herbs including *Punica granatum* (ii) Placebo	119	Double-blind, randomized clinical trial	Twice daily/8 weeks	No significant difference was observed in symptom and Quality of Life (QoL) scores between two groups.	[129]
Diarrhoea	(i) *Adansonia digitata fruit* (ii) Oral rehydration therapy	161	Prospective clinical trial	Daily with a follow-up after 4 to 48 h	The two treatments did not differ significantly in terms of diarrhoea duration and weight gain	[130]
Acute infective diarrhoea	(i) Berberine tannate (ii) Sulfadimidine and neomycin	55	Randomized clinical trial	Initial dose 40 mg followed by 20 mg every 4 h for 5 days	Faster recovery using berberine compared to standard antibiotic therapy with a clinical cure 72%	[103]
Antibiotic-associated diarrhoea (AAD)	(i) Berberine	63	Prospective study	NR	Cure rates 84 and 92% for C. *difficile* and non-*C.* *difficile* AAD, respectively	[112]
Acute gastroenteritis and dysentery	(i) Berberine and its combinations with, chloramphenicol, streptomycin, sulphaimidine and iodochlorhydroxyqinoline (ii) Antibiotics only	129	Clinical trial	300 mg/day for 7 days	Combinations of berberine with antibiotics were more effective than berberine and antibiotics alone	[131]
Acute nonspecific diarrhoea	(i) Bismuth subsalicylate (ii) loperamide hydrochloride	94	An open-label parallel comparison study bismuth subsalicylate	4900 mg/day for 2 days	Loperamide was significantly effective for diarrhoea treatment than bismuth subsalicylate	[121]
Enterotoxigenic *E. coli* and *V. cholerae* diarrhoea	(i) Berberine sulphate (ii) Berberine sulphate plus tetracyclic (for Cholera patients) (iii) Tetracycline alone	165	Randomized clinical trial	400 mg or 1200 mg berberine sulphate in a single oral dose	Reduced mean stool volume during three consecutive 8-hr periods after treatment	[107]
Infectious gastroenteritis	(i) Phytodrug (QG-5^®^) developed from guava leaves, standardized in its content of quercetin (ii) Control	100	Randomized clinical trial	500 mg every 8 h for 3 days	Decreased the duration of abdominal pain with no significant changes in the consistency and frequency of liquid stools compared with the control group.	[101]

## 4. OMICS Applications for Studying Biological Effects of Herbal Products against GI Bacteria

Currently, omics approaches, which encompass genomics, transcriptomics, proteomics, and metabolomics, are becoming important in identifying and characterizing crucial gene–protein–metabolite networks, discovering new drug metabolites, identifying complete genomes, transcriptomes, and proteomes of medicinal plants, evaluating human cell responses to drugs and whole ethnobotanical plants for medicinal use, and facilitating large-scale production of plant-derived medicines [132,133]. A research strategy that is based on the study of complex interactions between active constituents utilizing “omics” technologies (transcriptomics, proteomics, and metabolomics) and systems biology will be imperative to developing effective herbal-based treatments for infectious diseases.

### 4.1. Metabolomics in Antimicrobial Medicinal Plant-Based Drug Discovery

The discovery of plant biosynthetic pathways and gene clusters has increased dramatically over the past few decades, spurred by technological advances in sequencing and mass spectrometry technologies [134]. Due to the functional diversity and structural complexity of biosynthetic pathways in plants, the expansion of biosynthetic pathway discovery in plants lags behind that of bacteria and fungi [135]. A wide range of plant biosynthetic pathways have been explored using metabolomics-based approaches, such as chromatography coupled with mass spectrometry. A variety of analytical and computational metabolomics approaches being developed in recent decades for profiling specialized metabolites and analysing biosynthetic pathways, plant-based natural product discovery remains challenging despite significant technological advances in instrumentation, software, and databases [136]. This is mainly because of two reasons. The first is the diversity of functional and chemical properties of specialized metabolites in plants. Secondly, the specialized metabolites produced by a plant are part of its total metabolome. Since classical reductive approaches (experimentation and targeted approaches) to characterizing metabolites are time-consuming and laborious, untargeted metabolomics using mass spectrometry (MS) can be used to profile specialized metabolites in crude extracts and identify unprecedented numbers of metabolic classes [134]. Identification of metabolites from a variety of sources, including leaves, roots, soils, and volatiles, has led to the identification of biosynthesis pathways by identifying key shifts in the metabolite profile. While full metabolite scans with chromatography and mass spectrometry (MS1 mode) are (more accurately) quantified, they are unreliable in metabolite annotation, since several compounds can have the same mass but have different molecular formulas or may have the same molecular formula but differ in their chemical structure [133,137]. To address this challenge, metabolites are further fragmented using tandem MS mode (also known as MS2 or MS/MS or MSn when deeper fragmentation levels are included). With the aid of a variety of software and tools that have been developed for mining and annotating MS2 fragmentation data, metabolites can be structurally annotated and readily identified [137]. Aside from developing new classes of antibiotics with direct growth inhibition effects against bacteria, future research should also focus on finding indirect antimicrobials that are more effective and have better safety profiles. To understand the mechanisms of action at a molecular level, it is crucial to investigate the metabolome of pathogens after treatment.

Metabolomics experiments offer a more efficient route to drug discovery than the classical approach in natural products research. In essence, metabolomics analyses all metabolites present in a living system qualitatively and quantitatively [136]. Interestingly, this concept can be extended to the study of the relationship between whole metabolomes of natural products and their biological effects [134]. Different studies suggest that various enzymes, metabolic pathways, cell membrane or cell wall transport proteins and chaperones may also serve as potent targets for berberine, a natural product isolated from medicinal plants [66,138,139,140,141]. In a metabolomics study which combined principal component analysis, the antibacterial activity of traditional Chinese medicine *Aquilegia oxysepala* extract on *Staphylococcus aureus* and its main chemical components (genkwanin, apigenin, maguoflorine, and berberine), which are used for treating GI infections, were examined in comparison with nine antibiotics with known mechanisms of action [142]. As shown by this study, *A. oxysepala* targets are similar to those of protein synthesis inhibitors including lincolmensin, erythromycin, chloromycetin, streptomycin, and acheomycin, and its bioactive component maguoflorine accounted for most of the antibacterial activity. In this study, berberine showed similar effects to rifampicin and norfloxacin, both of which target nucleic acids [142]. These metabolomics strategies, which compare phytochemicals with known antibiotics, may be somewhat limited since they do not have the ability to define a new mechanism of action, but are viable and unexplored options for tagging new bioactive compounds with known mechanisms.

### 4.2. Transcriptomics and Functional Analysis of Proteins in Medicinal Plants

Transcriptomics is an effective method for assembling genomic data from a variety of non-model therapeutic plants that lack a reference genome. Transcriptomic studies assist in understanding the molecular mechanisms involved in the formation of secondary metabolites and in probing pharmaceutically significant mechanisms [143]. The transcriptomic analysis of *Catharanthus roseus* (L.) G. Don (Madagascar Periwinkle) revealed diverse iridoid-based monoterpene indole alkaloids [144]. It was determined from these transcriptomic data that a new iridoid synthase was responsible for converting 10-oxogeranial into iridoid scaffold [145] and that cytochrome P450 hydroxylation genes are involved in the biosynthesis of monoterpenoid indole alkaloids [146]. There are a number of non-glycosidic iridoids that possess strong antibacterial properties, including mussaenin A, gardendiol, isoboonein, and rehmaglutin D, which have a similar activity to chloramphenicol against *E. coli* [147].

A wide range of functions can be achieved with proteomics in medicinal plant research [148]. Using proteomics, we can visualize how proteins are structured, function, and modified, as well as how they interact with each other in vitro and in vivo [143]. Proteomics is a multi-functional field that can help predict the protein targets of plant-based bioactive compounds. It can also be used to understand how traditional Chinese medicine (TCM) acts on bacterial cells and how protein–drug interactions occur at the molecular level [149].

The proteomic analysis of *Salmonella enterica* revealed that energy metabolism-related proteins and flagellar proteins were up-regulated with berberine treatment, indicating a possible antibacterial action at the molecular level [150]. Although berberine is a cationic alkaloid, its water solubility is poor, which explains its reduced intestinal absorption. In animal models, it was observed that berberine’s bioavailability was much lower than 1%, since the amount that is absorbed by the gut is excreted back into the colon by P-glycoprotein [127].

These studies show how OMICS technologies have potential for identifying the mechanisms of action of bioactive compounds, as well as combating drug-resistant bacteria. Furthermore, these studies suggest that bacterial cell death cannot be explained solely by the direct interaction between bioactive compounds and their cellular targets [151,152], with several reports indicating that bacterial metabolic states can affect antibiotic effectiveness [153,154].

## 5. Safety Implications Regarding the Use of Plant-Derived Antimicrobials

The appeal of herbal medicine is multifactorial. It is widely believed by the general public that herbal medicines are safe to consume and are not associated with any significant side effects. Since these treatments are “natural”, they are thought to be safe as an alternative to conventional medicine. However, several side effects have been reportedly associated with herbal products used in the treatment of GI disorders. These are listed in Table 3. Further, herbal products are rarely subject to the same rigorous regulations as applied to other non-herbal medicinal products [155]. In the European Union (EU), herbal products are either categorized as food supplements or medicines; those which are “Classified as Medicine” are regulated under EU medicinal law Directive 2004/24/EC. The European Medicines Agency (EMA) handles the safety, efficacy, and premarket authorisation of these products. However, in the United States, the Food and Drug Administration (FDA) considers herbal products as dietary supplements and as such, they do not need to be registered with the FDA. The regulatory landscape in other countries has been summarised elsewhere [126].

## 6. Future Perspectives

Many previous studies concerning plant-derived antibacterial agents failed to standardize their methods to achieve reproducible and comparable results. The effectiveness of a plant’s antimicrobial properties is generally demonstrated through large-scale, well-designed clinical trials. It is therefore essential that clinical trials follow the guidelines of the U.S. FDA and the EMA when evaluating medicines indicated for treating bacterial infections [180,181]. Plant-derived products should also be evaluated in vitro for their antimicrobial effectiveness according to the methods recommended by the Clinical and Laboratory Standards Institute (CLSI) or by the European Committee on Antimicrobial Susceptibility Testing (EUCAST) [182].

As bacteria become increasingly resistant to commonly used antibiotics, new classes of plant-derived antimicrobials to treat infections caused by pathogens, such as *C. difficile, Enterobacter*, *E. coli*, *K. pneumonia*, and *Pseudomonas*, are of intense interest. The potential synergy or potentiation of phytochemicals with other antibacterial agents has already been demonstrated in previous studies [14,63,183]. In this sense, the use of plant constituents in the development of resistance-modifying drugs, such as agents affecting membrane permeability or modified antimicrobial targets, as well as agents affecting bacterial enzymes, inactivating antibiotics or efflux pumps, is becoming an area of active research [184].

As part of their defence against microbial infections, plants use a variety of chemical strategies to reduce the selective pressure for developing antibiotic resistance. The activity of a compound can be significantly increased or decreased depending on its synergistic and antagonistic effects with other compounds found in plants. The complex chemical composition of plant extracts may also produce pharmacological effects, as is the case for traditional herbal medicines, whose efficacy is based on their complex composition, due to their synergistic or antagonistic interactions with a wide spectrum of phytochemicals [63,185]. Developing more effective plant-derived antibacterial agents seems to be possible by developing chemically standardized complex phytotherapeutic preparations with a balanced ratio of antimicrobial, synergistic, and solubility-enhancing constituents. Nevertheless, there are specific problems associated with the use of complex phytotherapeutics as well as those associated with single-ingredient plant drugs, such as a lack of standardization, safety, and effectiveness validation, quality control, and the lack of patent rights over herbal medicines [186] In order for herbal products to succeed, clear intellectual property rights and standardized chemical and quality control standards must be established.

## 7. Conclusions

This review summarizes data on the clinical efficacy and antimicrobial properties of antibacterial agents of plant origin currently used to prevent and treat GI infections. A paradigm shift has occurred from focusing on conventional antibiotic therapy to alternative approaches due to new resistant bacterial strains. There is a wide variety of active constituents found in plants, making them an excellent source of antimicrobial agents with therapeutic potential as alternatives or potentiators of antibiotics. The identification of new and valuable antibacterial compounds from plants and the testing of their antibacterial properties are crucial for this purpose. Several factors may contribute to the effectiveness of herbal-derived compounds for fighting bacterial infections, two of which may be pivotal. First, from a phytochemical point of view, these compounds belong to various classes of plant secondary metabolites such as isoquinoline alkaloids, monoterpenoids, phenolic acids, and tannins, which may provide alternative antibacterial mechanisms to conventional antibiotics. Second, it is extremely likely that the utilization of unique traditional knowledge of herbal medicine will result in biocompatible, cost-effective, and promising solutions, ultimately leading to the discovery of new antibacterial agents. Although new plant-derived antibacterial compounds are increasingly being studied, few are still in clinical trials. However, most of them show antibacterial activity in vitro and in vivo. Most of these products act as direct growth inhibitors; however, certain products have antimicrobial properties due to their antitoxin properties (e.g., green tea extract). Inhibitory agents of biofilm formation may also be of interest. Plant-derived products may be used as biofilm inhibitors and synergistic agents in the future. It may be more fruitful to investigate combinations of plant compounds with conventional antibiotics. There may be a reason for this, since phytomedicines require complex combinational effects between their bioactive compounds to synergize their activities. Innovative approaches using high-throughput technologies (omics) and systems biology are crucial for effective proof-of-concept research and development of new types of plant-derived products that could be effective against antimicrobial resistance.

## Figures and Tables

**Table 1:** Mechanism of action of plant-derived substances (PDSs) against a broad range of GI microbes.

Phytochemicals	Chemical Structure	Plant	Mode of Action	Microorganism	(s)
Proanthocyanidin	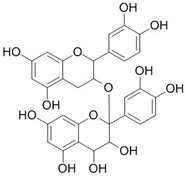	*Vaccinium macrocarpon* L.	Modifies biofilm formation	*Enterococcus faecalis, E. coli*	[25,26]
Zingerone	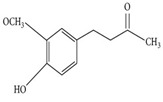	*Zingiber officinale* Rosc.	Reduce heat-labile enterotoxin (LT)-induced diarrhoea in ETEC through blocking the binding to GM1 ganglioside receptors	ETEC	[27]
Epigallocatechin	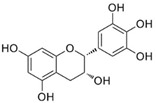	*Camellia sinensis* L.	Inhibit extracellular release of Vero toxin from enterohemorrhagic *Escherichia coli* O157:H7	EHEC	[28]
Quercetin	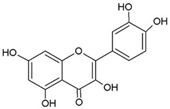	*Allium cepa* L.	Inhibition of ATPase activity, elevates extracellular phosphatase and galactosidase.	*E. coli*	[29,30]
Berberine	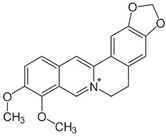	*Berberis vulgaris* L.	Targeting proteins responsible for upholding the structure of cells and for cell division	*E. coli, Salmonella* spp., *V. cholerae*	[31,32]
Eugenol	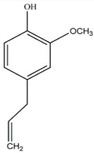	*Syzygium aromaticum* L.	Inhibition of toxin production	EHEC	[33]
Cinnamaldehyde	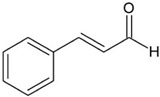	*Cinnamomum verum* J. Presl	Inhibition of toxin genes and host receptor expression, reducing toxin-mediated pathology	EHEC, *C. difficile*	[33,34]
Baicalin (5,6,7-trihydroxyflavone)	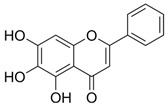	*Scutellaria baicalensis* Georgi.	Inhibition of toxin production	*H. pylori*	[35,36,37,38]
Thymol	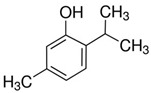	*Thymus vulgaris* L.	Disturbance of the cell membrane and cytoplasm	*C. difficile*	[39]
Geraniol	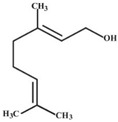	*Monarda fistulosa* L.	Disturbance of the cell membrane and disruption of the cytoplasm.	*C. difficile*	[39]
Pyrrolizidine	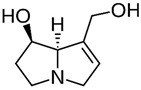	*Alkanna tinctoria* L.	Disturbance of the cell membrane and cytoplasm	*E. coli*	[40]
Martine	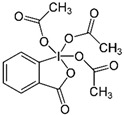	*Sophora flavescens* Ait.	Inhibiting the synthesis of proteins	*E. coli*	[41]
Isothiocyanates	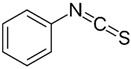	*Brassica oleracea* var. botrytis	Acting on cell membranes and leakage of cellular metabolites	Pathogenic *E. coli* strains	[42]
Andrographolide	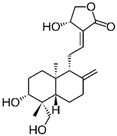	*Andrographis paniculata* (Burm.f.) Nees	Anti-secretory activity against enterotoxins (Heat Labile (LT) and Heat Stable (ST) forms)	Pathogenic *E. coli*	[43]
α-bisabolol	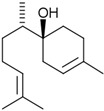	*Matricaria chamomilla* L.	Inhibition of efflux pump	*E. coli*	[44]

*EAEC* Enteroaggregative *Escherichia coli*, EHEC Enterohemorrhagic *Escherichia coli*.

**Table 3:** Toxicological effects of selected plant species used for the treatment of different GI disorders.

Plant Species	Common Name	Family	GI Disorder(s)	Toxicological Effects
*Psidium guajava* L.	Guava	Myrtaceae	Acute diarrhoea [96,156]	Minor liver inflammation in rats, LD50 = 1000 mg/kg [102]
*Musa × paradisiaca* L.	Plantain	Musaceae	Infectious diarrhoea [157]	Significant changes in white blood cells, eosinophils, platelets, neutrophils, and monocytes count [158].
*Leea indica* (Burm.f.) Merr.	Bandicoot berry	Vitaceae	Diarrhoea, dysentery [159]	Liver toxicity in rats [160]
*Acorus calamus* L.	Sweet flag	Acoraceae	GI infections, diarrhoea, dysentery [161,162]	Acute liver, spleen, and kidney toxicity and genotoxic effects in rats, LD50 = 221 g/kg [161]
*Cassytha filiformis* L.	Love-vine	Lauraceae	Diarrhoeagenic bacterial infections [163]	Acute haematological and biochemical toxicity (significant increase in alanine aminotransferase (ALT), aspartate aminotransferase (AST), and total and direct bilirubin in rats, LD50 = 625.8 g/kg [164])
*Zingiber officinale* Roscoe	Ginger	Zingiberaceae	Gastric ulceration, flatulence, diarrhoea [27,165]	Embryo toxic to pregnant rats [166]
*Thunbergia laurifolia* Lindl.	Laurel clock vine	Acanthaceae	Gastric ulcer, diarrhoea [156]	Decrease red blood cells in male rat [167]
*Senna occidentalis* (L.) Link	Coffee senna	Fabaceae	Constipation, GI infections [168]	Intestinal disturbance in long term use [169]
*Senna alata* (L.) Roxb.	Candle Bush	Fabaceae	Constipation, abdominal pain [168,170]	Decrease haemoglobin and erythrocyte (RBC) count values in rats [171]
*Euphorbia hartal* L.	Hairy Spurge	Euphorbiaceae	Diarrhoea, dysentery, constipation, intestinal parasites [172]	Leucocytosis, dullness, anorexia, stairy haircoat and 20% mortality in rats [173]
*Euphorbia heterophylla* L.	Milkweed	Euphorbiaceae	Intestinal bacterial infections, diarrhoea [172,173]	Leucopaenia in rats [173]
*Kaempferia parviflora* L.	Thai ginseng	Zingiberaceae	Flatulence, gastric ulcer [156,174]	Hepatotoxic to rats [175]
*Flemingia macrophylla* (Willd.) Kuntze ex Merr.	Apa apa	Fabaceae	Flatulence, indigestive [156]	Severe hypoglycaemia followed by death within 24 h after administration to normoglycemic mice [176]
*Celastrus paniculatus* Willd.	Black oil plant	Celastraceae	Diarrhoea, gastric ulcer, bowel spasms [177,178]	Hyperactivity and loss of behavioural responsiveness (loss of righting reflex in rat) [179]

## Data Availability

Not applicable.
